# Advances in methods to analyse cardiolipin and their clinical applications

**DOI:** 10.1016/j.trac.2022.116808

**Published:** 2022-12

**Authors:** Javier S. Bautista, Micol Falabella, Padraig J. Flannery, Michael G. Hanna, Simon J.R. Heales, Simon A.S. Pope, Robert D.S. Pitceathly

**Affiliations:** a Department of Neuromuscular Diseases, UCL Queen Square Institute of Neurology, London, UK; b Neurometabolic Unit, The National Hospital for Neurology and Neurosurgery, London, UK; c Neurogenetics Unit, Rare and Inherited Disease Laboratory, North Thames Genomic Laboratory Hub, London, UK; d NHS Highly Specialised Service for Rare Mitochondrial Disorders, Queen Square Centre for Neuromuscular Diseases, The National Hospital for Neurology and Neurosurgery, London, UK; e Genetics and Genomic Medicine, UCL Great Ormond Street Institute of Child Health, London, UK

**Keywords:** Cardiolipin, Lipids, Mitochondria, Liquid chromatography, Mass spectrometry, Clinical analysis

## Abstract

Cardiolipin (CL) is a mitochondria-exclusive phospholipid, primarily localised within the inner mitochondrial membrane, that plays an essential role in mitochondrial architecture and function. Aberrant CL content, structure, and localisation have all been linked to impaired mitochondrial activity and are observed in the pathophysiology of cancer and neurological, cardiovascular, and metabolic disorders. The detection, quantification, and localisation of CL species is a valuable tool to investigate mitochondrial dysfunction and the pathophysiological mechanisms underpinning several human disorders. CL is measured using liquid chromatography, usually combined with mass spectrometry, mass spectrometry imaging, shotgun lipidomics, ion mobility spectrometry, fluorometry, and radiolabelling. This review summarises available methods to analyse CL, with a particular focus on modern mass spectrometry, and evaluates their advantages and limitations. We provide guidance aimed at selecting the most appropriate technique, or combination of techniques, when analysing CL in different model systems, and highlight the clinical contexts in which measuring CL is relevant.

## 1 Introduction

**Fig. 1:**
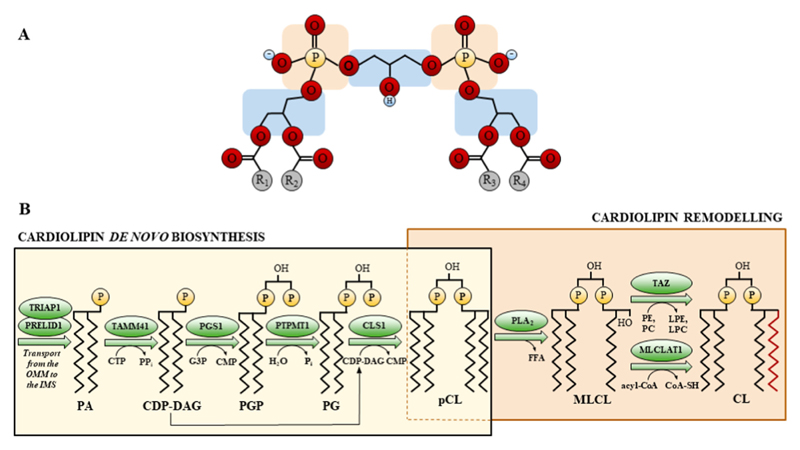
Overview of the chemical structure and biosynthetic pathway of cardiolipin (CL) (A) Chemical structure of mature CL containing two phosphate backbones (orange), three glycerol moieties (blue) and four fatty acyl chains (R1-R4), which can take different lengths or degrees of saturations. (B) Biosynthesis and remodelling pathways for CL. Abbreviations: Acyl-CoA, acyl-coenzyme A; CDP-DAG, cytidine diphosphate-diacylglycerol; CL, cardiolipin; CLS1, CL synthase; CMP, cytidinemonophosphate; CoA-SH, coenzyme A (unconjugated); CTP, cytidinetriphosphate; FFA, free fatty acid; G3P, glycerol-3-phosphate; H_2_O, water; IMS, intermembrane mitochondrial space; LPC, lyso-phosphatidylcholine; LPE, lyso-phosphatidylethanolamine; MLCL, monolysocardiolipin; MLCLAT1, monolysocardiolipin acyltransferase 1; OMM, outer mitochondrial membrane; PA, phosphatidic acid, PC, phosphatidylcholine; pCL, premature CL; PE, phosphatidylethanolamine; PG, phosphatidylglycerol; PGP, phosphatidylglycerol phosphate; PGS1, phosphatidylglycerol phosphate synthase; P_i_, inorganic phosphate; PLA_2_, phospholipase A2; PP_i_, inorganic pyrophosphate; PRELID1, PRELI Domain Containing 1; PTPMT1, protein-tyrosine phosphatase mitochondrial 1; TAMM41, TAM41 mitochondrial translocator assembly and maintenance homolog; TAZ, tafazzin; TRIAP1, TP53 regulated inhibitor of apoptosis 1.

Cardiolipin (CL; 1,3-bis(sn-3’-phosphatidyl)-sn-glycerol) is the signature phospholipid of mitochondria and is primarily found in the inner mitochondrial membrane (IMM). It has a conical shape with a double glycerophosphate backbone, four fatty acyl (FA) side chains, and three chiral centres, arranged in three distinct glycerol moieties [1] (Figure 1A). CL constitutes approximately 15–20% of the total phospholipid content in mitochondria [2] and has been associated with a wide range of essential functions [3,4], including bioenergetics (i.e., the stabilization of the respiratory chain complexes and supercomplexes organisation [5,6]), mitochondrial dynamics [7], membrane architecture [7], apoptosis [8,9], mitophagy [10], and protein import [11]. In mammals, *de novo* CL biosynthesis is a multi-step process that occurs within the IMM and involves phosphatidic acid (PA), which is transported into mitochondria from the endoplasmic reticulum, and several enzymes, including: TAM41 mitochondrial translocator assembly and maintenance homolog (TAMM41); phosphatidylglycerol phosphate synthase (PGS1); protein-tyrosine phosphatase mitochondrial 1 (PTPMT1); and CL synthase (CLS1), which catalyses the formation of premature CL (pCL) [12]. pCL is remodelled by phospholipase A2 (PLA_2_) into the transient intermediate phospholipid monolysocardiolipin (MLCL), which is subsequently re-acylated by tafazzin (TAZ) to generate mature CL, with its characteristic four acyl chains [12] (Figure 1B).

CL exists with a wide variety of acyl-chain compositions that differ in length and saturation, and alterations in the molecular conformation of CL provide insights into its impaired synthesis/remodelling and disease pathophysiology [13]. Studies exploring CL acyl chain composition in cultured cells and murine tissues confirm that CL composition is partially controlled by tetra-linoleic acid availability and, in most tissues, the tetra-linoleic form of CL(18:2)_4_ is the most abundant species [14–16]. Notably, the brain exhibits a diverse CL acyl chain profile comprised of longer FA side chains (i.e., 20:4 and 22:6), possibly resulting from a reduced FA 18:2 import across the blood-brain barrier and subsequent incorporation of long-chained FA.

Understanding the diversity of tissue-specific CL acyl chain composition is crucial for the development of analytical assays and to interpret alterations in CL species under pathological conditions. The clinical significance of CL measurement is clearly established in the pathomechanism of Barth syndrome (BTHS), a monogenic, ultrarare disorder caused by mutations in the TAZ gene that encodes a mitochondrial transacylase, involved in the remodelling of MLCL into mature CL [17,18]. Patients with BTHS suffer from cyclic neutropenia, skeletal and cardiac myopathies, and growth retardation. Biochemically, BTHS patients have elevated MLCL levels and MLCL/CL ratio, which is commonly used as a sensitive diagnostic marker [19–21]. This emphasises the utility of measuring different acyl-chain compositions and other CL-related phospholipids during the clinical evaluation of patients with suspected CL-related disorders. Despite the medical relevance of MLCL, the interplay between MLCL and CL is not fully understood. Recently, mutations in the *CLS1* and *TAMM41* genes have been identified to cause multi-system mitochondrial disease, further highlighting the clinical relevance of CL [22,23]. Increasing evidence links aberrant CL metabolism and content to human disease, supporting the importance of developing sensitive and specific high-throughput methods for CL analysis and quantification. Table 1 summarises human conditions associated with CL abnormalities, including neurological disorders [24], cancer [25], and cardiovascular and metabolic disorders [26].

CL is a pharmacological target for Elamipretide, a small molecule under investigation in several clinical trials reported to improve the mitochondrial respiratory function via CL binding and stabilization [27]. CL also has significant potential as a tissue-specific biomarker as exemplified by the presence of brain-specific CL species in the plasma of patients following cardiac arrest [28]. Thus, CL has potential to diagnose and monitor disease progression, in addition to measuring efficacy during clinical trials, for several disease states.

Detection and quantification of CL is a valuable tool for confirming the presence of mitochondrial dysfunction, characterising pathophysiological mechanisms of disease, and clinical diagnostics. CL assays require specificity, due to the high diversity of CL species, and sensitivity, given the low abundance of CL within the cellular lipidome. Consequently, in parallel to the quantitative analysis of CL, qualitative measurements are required to enable the distinction of individual sample components. Identification of the different CL species is an intricate task and different methods are available depending on the scope of the analysis. In this review, we summarise the techniques currently available for measuring CL and discuss the advantages and disadvantages for each method when attempting to characterise CL content, structure, and localisation. A primary focus is on recent advances in mass spectrometric analysis of CL. Finally, we highlight the clinical scenarios in which CL measurement has existing and potential importance.

## 2 Methods to detect and quantify cardiolipin

**Fig. 2:**
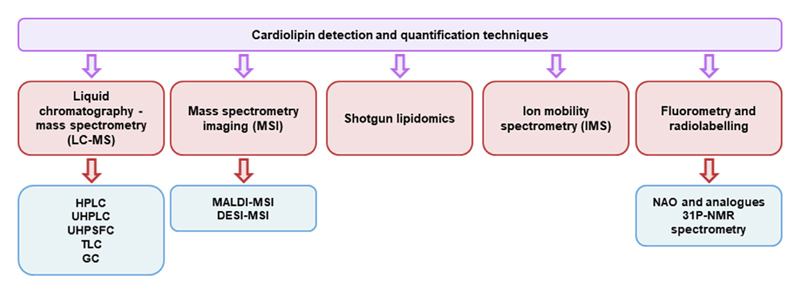
Schematic representation of available techniques for cardiolipin analysis. Fluorometric and radiolabelling techniques are not discussed in this review. Abbreviations: DESI-MSI, desorption electrospray ionisation mass spectrometry imaging; GC, gas chromatography; HPLC, high-performance liquid chromatography; IMS, ion mobility spectrometry; LC-MS, liquid chromatography-mass spectrometry; MALDI-MSI, matrix-assisted laser desorption/ionisation mass spectrometric imaging; MSI, mass spectrometry imaging; NAO, 10-N-Nonyl acridine orange; TLC, thin layer chromatography; UHPLC, ultra-high-performance liquid chromatography; UHPSFC, ultra-high-performance supercritical fluid chromatography; 31P-NMR, Phosphorus-31 nuclear magnetic resonance.

A wide range of methods are available to measure CL. However, the specificity and sensitivity vary according to the nature of biological sample, separation strategy, and detection technique. The different analytical strategies can be divided into five broad groups (Figure 2): 1) liquid chromatography, usually combined with mass spectrometry (LC-MS); 2) mass spectrometry imaging (MSI); 3) shotgun lipidomics; 4) ion mobility spectrometry (IMS); and 5) fluorometry and radiolabelling.

The focus of this review will be on mass spectrometric techniques, which are becoming widely available and have many advantages, in terms of sensitivity and specificity, over older chromatographic and fluorometric techniques. In recent years, LC-MS-based multi-analyte/lipidomic assays have also been introduced in clinical practice (see Ref. [29] for review).

### 2.1 Lipid extraction prior to analysis

Prior to measurement, biological lipids must be extracted and isolated. One exception is when fluorescent dyes are used, such as 10-N-Nonyl acridine orange (NAO), which can be applied directly to cells or tissue [30]. Extraction of lipids from biological samples is a crucial pre-analytical step to ensure optimal chromatographic separation of low abundant species, such as CL. Several lipid extraction methods have been described; however, the two most widely used are Folch (1957) and Bligh and Dyer (1959) protocols [31,32]. Both methods utilise chloroform and methanol in different ratios to partition sugar and proteins in the top aqueous phase, and lipids in the lower organic phase. The organic layer is subsequently removed, dried down, and resolubilised for analysis. Despite representing gold standard methods for lipid extraction, these two chloroform-based protocols present some disadvantages, including the use of a toxic and carcinogenic solvent (i.e., chloroform), and the presence of lipids in the bottom sample phase, which increases the risk of contaminating the lipid mixture during extraction. Various protocols using simpler lipid extraction techniques and fewer toxic solvents have therefore been developed. Among these, the methyl *tert*-butyl ether (MTBE) lipid extraction procedure has proved faster and safer than conventional methods [33]. In addition to lower toxicity compared with chloroform, the advantages of this method include good recovery for all lipid groups and the presence of an organic layer settled on top of the aqueous phase, thus enabling easier automation of the extraction procedure. One potential disadvantage is represented by water contamination of the organic phase, which results in longer drying down times and carryover of contaminant species that can cause ion suppression. Another extraction protocol is based on the BUME (Buthanol:Methanol) method, which uses butanol and methanol resulting in a single phase extraction [34]. The initial single phase extraction with Butanol:Methanol (3:1) is followed by addition of heptanol:ethyl acetate (3:1) and 1% acetic acid. The top organic layer contains the lipids and can be removed, dried down, and resuspended for analysis.

Solid phase extraction (SPE) has also been used as an additional clean-up step before phospholipid/CL analysis. Helmer and colleagues used a simple hydrophilic interaction liquid chromatography (HILIC)-based SPE cartridge method to clean up lipid extracts and look at oxidised CL (CLox) and MLCLs [35]. This method is described as quick and simple and it avoids the use of strong organic solvents, such as hexane, which are typically used for normal phase SPE of lipids.

### 2.2 Analytical techniques for CL analysis

Following extraction, complex lipids (e.g., CL) can be separated by either LC or thin-layer chromatography (TLC) [36], and the individual FA side chains analysed by gas chromatography (GC). These techniques are not described in detail here as they have largely been superseded by modern hyphenated mass spectrometric methods. Details of these techniques are summarised in Table 2.

#### 2.2.1 Liquid chromatography - mass spectrometry (LC-MS)

The sensitivity and accuracy of CL identification and quantification by chromatography has benefited from rapid advances in mass spectrometry. Most techniques involve separation of individual classes of lipid by LC followed by MS.

LC separation uses a non-polar or polar stationary phase and involves dissolution of the lipid mixture in a liquid mobile phase, enabling partitioning of single lipid components according to their polarity and molecular interactions with the stationary and mobile phase. The sample is injected and separated on a column with a densely packed stationary phase. A wide variety of different stationary phases, column parameters, and elution solvents are available. The separated compounds have a characteristic retention time and can be measured by the mass spectrometer as they are eluting from the column.

Lange et al. compared and described reversed-phase, normal phase, and HILIC for the separation of lipids [37]. Reversed phase is currently the most used technique for phospholipid separation; however, HILIC is becoming increasingly popular. HILIC separates the lipid mixture based on the polarity of the phospholipid head-group and has the advantage to have greater compatibility with electrospray ionisation techniques when compared to normal phase.

**Fig. 3:**
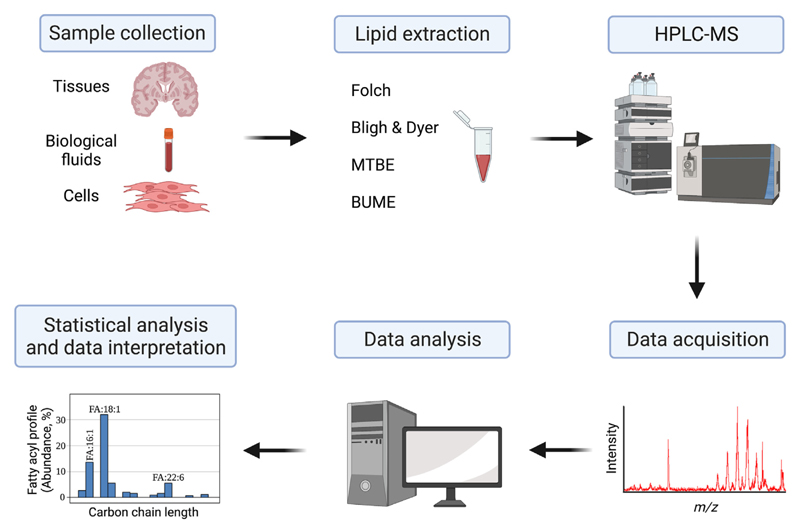
Steps involved in high-performance liquid chromatography mass spectrometry (HPLC-MS) of cardiolipin (CL) species, from sample collection to statistical analysis. Several sample types can be used, including tissues, biological fluids, and cell lines. Following lipid extraction, HPLC-MS acquires CL spectra, based on the identification, quantification, and mass of the lipid. Data is subsequently statistically analysed and interpreted. Figure created with BioRender.com. Abbreviations: BUME, Buthanol:Methanol; FA, fatty acids; HPLC-MS, high-performance liquid chromatography mass spectrometry; MTBE, Biphasic Methyl tert-butyl ether.

Figure 3 summarises the overall workflow to analyse CL by LC-MS, from sample collection to the statistical analysis. Several mass spectrometry ionisation and detection techniques are available. Following HPLC separation, the most commonly used ionisation method is electrospray ionisation (ESI). This is a ‘soft’ ionisation technique that enables ionisation of molecular species, without significant in-source fragmentation; singly or multiply charged ions are produced from the liquid eluate derived from the HPLC column. Many studies have used HPLC-ESI-MS to quantify CL in both singly and doubly charged states (see Refs. [21,38] for examples). The use of tandem mass spectrometry techniques has provided important structural information and improved the detection limits enabling analysis of lipids in the picomolar range.

‘Ultra-high-performance’ liquid chromatography (UHPLC) utilises smaller particles in the stationary phase and at higher pressure than HPLC. This enables faster and more sensitive measurements, thus facilitating higher sample throughput. The methodology for measuring CL with UHPLC has previously been described [39]. Importantly, UHPLC-MS is compatible with HILIC, thus supporting separation of polar metabolites [37]. Although this approach has been used for CL analysis [40], reversed-phase HPLC is usually used to separate CL, based on the hydrophobic nature of the FA side chains [14]. Normal phase chromatography has also been utilised and is recommended by some authors to prevent ESI matrix effects [41], although it can also suppress ionisation of analytes due to the nature of the organic solvents used. Table 2 summarises common LC-MS methods employed for the detection of CL in multiple biological samples.

HPLC-MS has been used to explore CL species as a diagnostic biomarker for human disease, with different pathologies presenting specific changes. For instance, decreased CL levels have been identified in patients with frontotemporal dementia (FTD, serum) [42], traumatic brain injury (TBI, brain tissue) [43], heart failure (cardiac tissue) [44,45], and hepatocellular carcinoma (tumour tissue) [46]. The specificity of HPLC-MS is particularly relevant when total CL content is normal, and the pathology is linked with aberrant levels of specific CL species or the ratio of CL with other phospholipids. For example, CL enriched with palmitoleic acid (CL-16:1) was detected in five of six patients with prostate cancer, and might explain the higher proliferation rates within the tumour cells [47]. Similarly, despite normal total CL levels, an increase in specific subspecies (CL-66:3, CL-66:4, CL-68:3, CL-68:4 and CL-68:5) in the fibroblasts of patients with MEGDEL syndrome [(3-methylglutaconic aciduria (MEG), deafness (D), encephalopathy (E), and Leigh-like disease (L)] is reported and confers potential as a biomarker [48]. Finally, the MLCL/CL_4_ ratio is increased in BTHS [19,49–52] and used as a diagnostic assay, further emphasising the advantages of applying mass spectrometry to study CL species. Once separated, they may be further fractionated by adsorption, ion-exchange chromatography, or by combinations of both.

Over the last decade, there has also been an increasing demand for methods to identify and quantify oxidised lipids (including CL), given their potential application as disease biomarkers. Reactive oxygen species (ROS) and reactive nitrogen species (RNS) readily modify the chemical structure of CL species and other phospholipids [53] due to the presence of unsaturated FAs. The reaction between ROS and CL leads to formation of CLox. The identification of CLox, and method standardisation, is a challenge because of the number and low abundance of such oxidised species. HPLC-MS has been crucial in elucidating mechanistical and clinical insights of CLox [54–57]. Importantly, CLox is implicated in the pathophysio-logical development of several conditions, including neurodegeneration, diabetes, myocardial infarction, and ageing [58]. Furthermore, HPLC-MS has facilitated the characterisation of cellular mechanisms that promote release of pro-apoptotic factors triggering the cell-death pathway; for example, the oxidation of polyunsaturated CL species by cytochrome *c* in fatally injured cells initiates the apoptotic process [59]. In addition, experimental TBI in rats leads to accumulation of more than 150 newly oxidised molecular species of CL that precipitate neuronal death [60]. CL oxidation has been also observed in experimental cerebral ischemia-reperfusion in rodents using 2D-LC-MS [61], and delivery of mitochondrially-targeted small molecule inhibitors, as electron scavengers, has shown to prevent accumulation of CLox products and the pathological consequences of brain injury. Thus, CL oxidation represents a target for neuro-drug discovery [60]. The effects of RNS on CL species and cellular functions remain largely unexplored. However, a new HPLC-MS method can detect nitroso, nitrated, and nitroxidised CL products, offering new opportunities to advance understanding in the pathophysiological consequences of RNS [62].

Finally, ultra-high-performance supercritical fluid chromatography (UHPSFC) has been suggested as a potential lipidomic method for CL measurement. UHPSFC uses a supercritical fluid, such as carbon dioxide, as a mobile phase. This allows low back pressure, high flow rates, and good solubility of lipids. UHPSFC has used to measure CL in porcine brain extracts [63]. A recent review of UHPSFC describes the benefits of this approach for speed of analysis and improved separation of non-polar lipids, compared to more traditional HILIC and reversed-phase chromatography [64].

#### 2.2.2 Mass spectrometry imaging (MSI)

CL abundance is relatively low in comparison with other cellular and tissue lipids. Consequently, there is limited understanding of the spatial distribution for CL using mass spectrometry in disease states; HPLC requires cellular homogenisation and precludes acquisition of spatial information.

In recent years, novel and label-free imaging techniques that are compatible with mass spectrometry have been developed to investigate mitochondrial content and acyl-chain composition, using a visualisation approach [65,66]. This has enabled mapping of lipid profiles and their contribution to cellular structure and pathology. Two major soft ionisation MSI techniques are used to visualise CL content: matrix-assisted laser desorption/ionisation mass spectrometric imaging (MALDI-MSI) and desorption electrospray ionisation mass spectrometry imaging (DESI-MSI).

Tissue imaging by MALDI-MSI is the most commercialised MSI technique. It offers label-free spatial resolution of diversified CL species and other phospholipids in tissues [65,67]. This tool allows the visualisation and mapping of diversified CL species across different brain areas and the structural MS/MS fragmentation and mapping of CL species, assuming use of a suitable mass detector. MALDI-MSI has shown a non-random distribution of individual oxidised and non-oxidised CL species across different brain tissues [67].

The selection and application of the matrix are critical for the absorption of the laser wavelength and the ionisation of lipids in the tissue sections. The optimisation of the most suitable MALDI matrix should be evaluated on the tissue sections and CL standards. Conventionally, 2,5-dihydroxy benzoic acid is the preferred matrix for lipid mapping and spectra using either spraying or sublimation protocols [65,68].

A variation of MALDI-MSI employs Fourier-transform ion cyclotron resonance (FTICR). MALDI-FTICR-MSI has been applied to interrogate different lipids in prostate cancer. Interestingly, the high intensity detection of specific CL species correlated with more severe phenotypes of tumour regions, suggesting CL as a promising biomarker for prostate cancer [69,70]. The technique has also been combined with time of flight mass spectrometry (MALDI-TOF-MSI) to measure CL [71]. This tool has been used to diagnose BTHS in patient-derived leukocytes, based on increased MLCL and pCL ratio over mature CL, and the changes in CL spectrum peaks in BTHS compared with healthy controls [72]. One advantage of this method is the simultaneous detection of CL and MLCL species using a single run of mass spectrometry analysis.

Another alternative technique is DESI-MSI, which uses an ion-isation technique that directly electrically charges the sample surface [73]. DESI-MSI can be performed at ambient operating conditions and with minimum sample preparations. However, this technique has a lower spatial resolution (30–50 μm) than MALDI-MSI (<10 μm) [74]. MALDI-MSI has shown increased content and chemical diversity of CL species in oncocytic thyroid tumors [75].

Finally, tandem use of MALDI-MSI and DESI-MSI has shown high spatial and mass resolution of CL species, and other phospholipids and gangliosides, detecting multiple analyte classes from tissue samples [76].

#### 2.2.3 Shotgun lipidomics

Shotgun lipidomics is the term used to described direct infusion mass spectrometry of lipid extracts without prior chromatographic separation [29,77]. Although it lacks the chromatographic resolution of LC-MS methods, when combined with a powerful mass spectrometer it can be used to undertake multiple mass spectrometry experiments in succession. It is generally used with high mass resolution mass spectrometers, including time of flight (TOF) and orbitrap mass spectrometers [29], and has been used for the analysis of CL species [29,77].

#### 2.2.4 Ion mobility spectrometry (IMS)

Owing to the variety and complexity of lipids and their side-chains, it is not always possible to unambiguously identify components by their mass and fragmentation patterns alone. Many species are isobaric and have identical atomic composition. To enable identification of such isobaric mixtures, technologies such as IMS have been introduced. IMS, a gas-phase electrophoretic technique, allows separation of these isobaric ions in the gas phase according to their shape, charge, and size. It therefore adds an additional dimension to the separation of lipid components. There are a variety of IMS technologies depending on the type of mass spectrometer being used [78]. Further details with regards to the use of IMS in lipidomics are given in a review by Paglia et al. [79]. Addition of field asymmetry ion mobility spectrometry (FAIMS) has been shown to enrich and increase the sensitivity for low abun-dance doubly charged CL species [80].

#### 2.2.5 Quantification of cardiolipin species by mass spectrometry

Quantification in mass spectrometry is usually performed using a stable isotope labelled internal standard with essentially identical chemical properties to the analyte of interest. This internal standard accounts for losses during sample processing and matrix suppression effects. The amount of analyte can be determined by the ratio of the analyte to internal standard and comparison to calibration curves. Quantification of CL and other lipids is complicated by the large number of species with different FA side chains present in each sample and the lack of appropriate stable isotope standards for each of these species. This is further complicated by the overlap of isotopic peaks (e.g. Mþ2) with those of CL species having saturated side chains (also an addition of two mass units for the loss of each double bond.). The problems and solutions to quantification of CL species are discussed in detail by Tatsuta [81]. The original CL detection method used a single non-naturally occurring internal standard (CL-(14:0)_4_). Other CL species are now available to be used as internal and external standards (e.g., CL mix by Avanti lipids). These mixed standards enable better quantification as they account for differences in chromatographic separation, matrix effects and ionisation efficiencies between CLspecies with different side chains. There is still a need to increase the availability (i.e., number and type) of CL standards. This is particularly important for studies of CLox species where no commercial standards are currently available.

Analysis and quantification of CL mass spectrometry data can be complex. Various proprietary (e.g. LipidView (SCIEX) [81]) and open source software (e.g. MZmine 2/3 [35]) have been used to aid the analysis of these data.

## 3 Clinical diagnostics

Several techniques are available for CL detection and/or measurement. However, the choice of the analytical method depends on the experimental question, level of detail, and sensitivity required. Table 3 summarises specific advantages and disadvantages of each method. Additional factors that influence this decision include the tissue or cell type available for analysis, the clinical and/or research question, and accessibility to the diagnostic facilities. Currently, few centres offer diagnostic CL analysis, and the focus is primarily aimed at diagnosing suspected BTHS using LC-MS. However, as the CL profile abnormalities linked with different human diseases have expanded, an urgent need to provide qualitative and quantitative diagnostic CL measurement has emerged. Examples of pathological states that would potentially benefit from detailed analysis of CL species include novel defects in CL synthesis and remodelling [22,82], mitochondrial disorders, FTD [42], TBI [43], cancer [46,47] and cardiovascular diseases [28]. Most of these disorders show changes in CL species in multiple biological tissues, which require modern mass spectrometric techniques for detection.

Several human biological samples can be utilised when investigating the role of CL in human health and disease, including cell lines (e.g., patient-derived fibroblasts), tissues (e.g., brain, liver, cardiac, and skeletal muscle), and biological fluids (e.g., blood, urine, and cerebrospinal fluid). In addition, induced pluripotent stem cells differentiated into disease-relevant cell types (e.g. cortical neurons, myotubes, or cardiomyocytes) enable researchers to characterise CL in a tissue-specific human disease model [83,84]. One important consideration is the choice of cell culture medium and growth conditions used, which may affect CL composition and mitochondrial function [16]. Importantly, high-end mass spectrometers are now increasingly available within diagnostic services. Consequently, any clinically relevant research findings are readily transferrable to diagnostic laboratories.

## 4 Conclusion and future perspectives

CL is emerging as a potential biomarker to diagnose and monitor disease progression, and as a potential pharmacological target, in several disease processes, including neurodegenerative disorders, cancer, cardiovascular and metabolic disorders. Despite advances in lipidomic techniques, challenges remain to ensure a complete understanding of CL metabolism is achieved. LC-MS and MSI techniques that assess individual phospholipid species have helped discern CL tissue-specificity and acyl chain composition. However, some of these methods are highly technical and require specialist equipment. Consequently, more cost-effective, scalable methods and/or probes that enable sensitive and reliable measures of CL would benefit diagnostic services, and selecting the most appropriate, disease-relevant assay is crucial. This may require the use of simplified, more targeted mass spectrometric assays, looking at subsets of CL species, in the clinical diagnostic setting. In future, characterising CL biosynthesis and remodelling pathways will provide additional insights into the pathophysiological implications of aberrant CL, while combining lipidomics and other state-of-the-art multiomics techniques will be required to fully appreciate the role of CL in human health and disease.

## Figures and Tables

**Table 1:** Cardiolipin abnormalities reported in human conditions.

Category	Condition	Biological sample	Cardiolipin abnormalities	Method	Ref.
Neurological	FTD	Serum from 40 FTD patients	~20% decrease of total CL levels	HPLC-MS	[42]
disorders	TBI	Brain tissue from the pericontusional area of 10 TBI patients	Increased CLox (1hr after TBI)	LC-MS/MS	[43]
			Increased MLCL by hydrolysis (4 and 24 h s after TBI) Decreased total CL Increased Taz expression (4 and 24 h s after TBI)		
	BTHS	Left and right heart ventricle tissue from two BTHS patient	Decreased L4CL	HPLC-MS	
		Skeletal muscle and platelets from two BTHS patients Platelets from six BTHS patients			
		PBMCs from five patients diagnosed with BTHS	Increase MLCL/CL ratio Decreased CD8^+^ T cells	HPLC-MS	[50]
		Fibroblasts from five patients diagnosed with BTHS	75% decreased CL pool size Decreased incorporation of linoleic acid	TLC	[82]
		Fibroblasts from five patients diagnosed with BTHS	13% decreased L4CL 12–25% decreased other CL species	HPLC-MS	[49]
		Blood from seven patients diagnosed with BTHS	Normal CL_4_ concentrations Increased MLCL/CL_4_ ratio	HPLC-MS/MS	[52]
		Leukocytes from 24 healthy donors and eight BTHS-affected boys	Decreased mCL Increased (MLCL þ pCL)/mCL	MALDI-TOF-MSI	[72]
			Increased compositional distances of CL fingerprints in BTHS patients.		
	MEGDELs yndrome	Fibroblasts from five patients diagnosed with MEGDEL syndrome	Unaltered total CL level Increased CL-66:3, CL-66:4, CL-68:3, CL-68:4 and CL-68:5	HPLC-MS	[48]
Cancer	HCC	Tumour tissues during HCC progression from 46 patients	Decreased total CL Decreased L_4_CL, ratio L_4_CL/total CL Decreased CLox Decreased PUFA, especially LA	HPLC-MS	[46]
	Prostate cancer	Prostate tissue cancer from 10 patients	Increased CL	MALDI-FTICR-MSI	[69]
		Prostate tissue cancer from three patients	Identification of 14 exclusive CL species in the cancerous region vs two exclusive CL species in non-cancerous regions	MCAEF-MALDI-FTICR-MSI	[70]
		Prostate tissue cancer from six patients	Increased palmitoleic acid (CL-16:1) within the CL molecules of five patients	HPLC-MS	[47]
	Thyroid oncocytic tumors	Oncocytic thyroid tumour tissues from 10 patients and non-oncocytic thyroid tumour tissues from 10 patients	Increased content and chemical diversity of CL species	DESI-MS	[75]
Cardiovascular and metabolic disorders	HF	Cardiac tissue from 10 patients diagnosed with idiopathic dilated cardiomyopathy	Decreased L_4_CL Increased minor CL species Decreased CL mass Constant CLox	HPLC-MS	[44]
		Left ventricular tissue from 21 LVAD-supported hearts	Decreased CL content LVAD-supported hearts recovered normal CL ratios in ischemic cardiomyopathy patients	HPLC-MS	[45]
	SVHF	Right ventricle myocardial tissue from 22 children (younger than 18 years) diagnosed with SVHF	Decreased total CL content Normal L_4_CL levels	HPLC-MS	[38]
	Cardiac arrest	Plasma from 39 patients resuscitated after cardiac arrest	Nine out of 26 brain-specific CL species found in plasma	HPLC-MS/MS	[28]
	TD	Fibroblasts from 3 homozygous TD patients	3–5 fold increased CL and MLCL	TLC	[85]
Others	Pneumonia	Tracheal aspirates from 17 patients diagnosed with pneumonia	~9.7 fold increased CL	NAO	[86]
	Ageing	Epidermal cells from 43 women with ages ranging 9- to 75-year-old	57% decreased CL levels	NAO	[87]

Abbreviations: BTHS, Barth syndrome; CL, cardiolipin; CLox, oxidised CL; DESI-MSI, desorption electrospray ionisation mass spectrometry imaging; FTD, frontotemporal dementia; HCC, hepatocellular carcinoma; HF, heart failure; HPLC-MS, high-performance liquid chromatography; L_4_CL, tetralinoleoyl cardiolipin; LA, linoleic acid; LVAD, Left Ventricular Assist Device MALDI-FTICR-MSI, Matrix assisted laser desorption/ionisation Fourier transform ion cyclotron resonance mass spectrometry imaging; MALDI-TOF-MSI, Matrix assisted laser desorption/ionisation time of flight mass spectrometry imaging; MCAEF, matrix coating assisted by an electric field; mCL, mature CL; MLCL, monolysocardiolipin; MEGDEL, [(3-methylglutaconic aciduria (MEG), deafness (D), encephalopathy (E), and Leigh-like disease (L)]; NAO, 10-Nonyl acridine orange; pCL, premature CL; PUFA, polyunsaturated fatty acids; SVHF, single right ventricle congenital heart disease; TAZ, tafazzin; TBI, traumatic brain injury; TD, Tangier Disease; TLC, thin-layer chromatography.

**Table 2:** Key methodologies used for cardiolipin (CL) analysis by liquid chromatography mass spectrometry (LC-MS).

Purpose of CL analysis	Lipid extraction details	CL LC-MS methodology	Quantification of CL species	Ref.
Bloodspot analysis for BTHS diagnosis using High resolution mass spectrometry	Blood spots extracted using 1 mL methanol/chloroform (1:1, vol/vol)	Column - Reversed phase Acquity HSS T3, 2.1 x 100 mm, 1.8 μm particle size Mass spectrometer - Q Exactive plus (Thermo Fisher Scientific, Waltham, MA) negative ion mode Buffers - Gradient - A (H_2_O/Methanol = 6/4 + 10 mM ammoniumformate +0.1% formic acid) and B (isopropanol/Methanol = 9/1 + 10 mM ammoniumformate +0.1% formic acid)	CL(14:0)_4_ was used as an IS. Ratio of MLCL/CL was measured using peak areas of extracted ion chromatograms of exact masses in scan mode. Estimated concentrations of CL species was calculated by dividing peak area by that of IS and multiplying by the concentration of IS. Analysing singly charged CL and MLCL species reduced background noise and improved sensitivity.	[21]
Analysis of CLox species and oxidised FA to study the role of CL oxidation	Folch extraction of brain tissue	Column - Normal phase Luna 3 μm Silica, 100 Å, 150 x 2 mm Mass spectrometer - Q-Exactive plus hybrid Quadrupole-Orbitrap mass spectrometer (Thermo Fisher Scientific) - negative ion mode Buffers - Multistep gradient. A (hexane/propanol/water/triethylamine/formic acid, 43:57:1:0.5:0.01 v/v containing 10 mM ammonium acetate) and B (hexane propanol/water/triethylamine/formic acid, 43:57:1:0.5:0.01 v/v containing 10 mM ammonium acetate) Free fatty acids determined by reversed phase LC-MS	CL(14:0)_4_ was used as an IS. MS data analysed using SIEVE software (Thermo Fisher Scientific) using an in-house database of all CL and oxidised CL species. Exact masses were used for quantification and concentrations calculated against a calibration curve of IS vs CL(18:2)_4_.	[43]
Stable isotope labelled linoleic to trace CL synthesis in T cells	MTBE extraction from blood cells	Column - Reversed phase Zorbax Eclipse plus C18 column (100 x 2 mm, 1.8 μm particles) Mass spectrometers - Agilent 6495 Triple Quad QQQ-MS and Bruker Impact II QTOF-MS operating in negative ion mode. Buffers - gradient of buffer A (10 mM ammonium formiate in 60:40 acetonitrile:water) to 97% buffer B (10 mM ammonium formiate in 90:10 2- propanol:acetonitrile)	Agilent Mass Hunter software used to identify and quantify lipids by fragmentation and retention time. CL was quantified in linoleic acid and glucose tracer experiments using a QTOF-MS. CL species > M+4 were used. An in-house script was used to correct for natural abundance.	[50]
CL and precursor lipids to study full synthetic pathway	Fibroblasts and heart tissues extracted using 1 mL of 1-butanol and 500 μL of water-saturated 1-butanol	Column - HILIC silica column (50 mm x 2.1 mm), with a 2.6 μm particle size Mass spectrometer - A hybrid triple quadrupole linear ion trap mass spectrometer API 4000 Q-Trap in the positive ESI mode Buffer – A (0.2% formic acid and 200 mM ammonium formate) and B (acetonitrile containing 0.2% formic acid)	CL(14:0)_4_ was used as an IS. Quantification was performed using MRM transitions using the loss of DAGs. Symmetric CL species lose a single DAG specie. Asymmetric CL species lose two different DAGs so are multiplied by 2 for quantification purposes. A six point calibration curve was performed using two different CL species, one symmetric and one asymmetric. Correction for isotopic overlap was performed and described in detail.	[34]

Abbreviations: BTHS, Barth syndrome; CL, cardiolipin; m/z, mass-to-charge ratio; CLox, oxidise CL; DAG, diacylglycerol; ESI, electrospray ionisation; FA,fatty acids; HILIC, Hydrophilic-interaction chromatography; HSS, High Strength Silica; IS, internal standard; MLCL, monolysocardiolipin; MRM, Multiple reaction monitoring ; MTBE, Biphasic Methyl *tert*-butyl ether; PUFA, polyunsaturated fatty acids; QTOF-MS, quadrupole time of flight mass spectrometry; TBI, traumatic brain injury.

**Table 3:** Advantages and disadvantages of different techniques available to detect and quantify cardiolipin.

Technique of quantification	Advantages	Disadvantages	Qualitative information?
LC-MS	High throughput screenings, with high sensitivity and selectivity Best established method	Disregard cellular heterogeneity and spatial localisation due to cellular homogenisation Expertise required for interpretation	Yes
TLC	Inexpensive and easy to use Different stainings can be performed Separation of complex mixtures possible	Separation is limited to the length of the plate Limited reproducibility	No
GC	Highly established in fatty acids analysis Best for the separation of volatile samples	Derivatisation of the analyte is required to make the compounds volatile Limited to thermally stable and volatile compounds	Yes
MALDI-MSI	Lipid extraction and separation steps may not be required Direct analysis of biological samples Visualisation and mapping of different CL species across brain tissues	Low ionisation efficiencies of small molecules Complex sample preparation for large molecules The matrix can create suppression effects interfering with low mass-to-charge (*m/z*) analytes	Yes
DESI-MSI	Analysis of CL spatial distribution in biological samples Direct analysis of the tissue samples Open air environment Minimal sample preparation	The efficiency of ionisation droplet formation can be affected by the matrix Challenging experimental automatisation and reproducibility	Yes
Shotgun lipidomics	High reproducibility No need of prior chromatographic separation	Lower chromatographic resolution than LC-MS methods	Yes
IMS	Improved separation and identification of CL species	Highly specialist technique and not widely available	Yes
NAO	Cheap and easy-to-use probe Convenient and rapid method to perform in the lab	Conflict in academia regarding the reliability of NAO Not recommended for clinical samples	No

Abbreviations: CL, cardiolipin; DESI-MSI, desorption electrospray ionisation mass spectrometry imaging; GC, gas chromatography; HPLC-MS, high-performance liquid chromatography; IMS, ion mobility spectrometry; LC-MS, Liquid chromatography-mass spectrometry; MALDI-MSI, Matrix-assisted laser desorption/ionisation mass spectrometry imaging; NAO, 10-Nonyl acridine orange; TLC, thin-layer chromatography.

## Data Availability

No data was used for the research described in the article.

## Abbreviations

BTHS: Barth syndrome
CL: cardiolipin
CLox: oxidative
CL; DESI-MSI: desorption electrospray ionisation mass spectrometry imaging
FA: fatty acyl
HPLC-MS: high-performance liquid chromatography-mass spectrometry
IMM: inner mitochondrial membrane
IMS: ion mobility spectrometry
MALDI-MSI: matrix assisted laser desorption/ionisation mass spectrometry imaging
MLCL: mono-lysocardiolipin
NAO: 10-N-Nonyl acridine orange
pCL: premature cardiolipin
ROS: reactive oxygen species
SPE: solid phase extraction
UHPLC: ultra-high-performance liquid chromatograph
UHPSFC: ultra-high-performance supercritical fluid chromatography
